# The lubrication performance of the ceramic-on-ceramic hip implant under starved conditions

**DOI:** 10.1016/j.jmbbm.2015.06.001

**Published:** 2015-10

**Authors:** Qingen Meng, Jing Wang, Peiran Yang, Zhongmin Jin, John Fisher

**Affiliations:** a Institute of Medical and Biological Engineering, School of Mechanical Engineering, University of Leeds, UK; b School of Mechanical Engineering, Qingdao Technological University, China; c School of Mechanical Engineering, Xi׳an Jiaotong University, China

**Keywords:** Ceramic-on-ceramic, Hip implant, Lubrication, Starvation, Tribology

## Abstract

Lubrication plays an important role in the clinical performance of the ceramic-on-ceramic (CoC) hip implant in terms of reducing wear and avoiding squeaking. All the previous lubrication analyses of CoC hip implants assumed that synovial fluid was sufficiently supplied to the contact area. The aim of this study was to investigate the lubrication performance of the CoC hip implant under starved conditions. A starved lubrication model was presented for the CoC hip implant. The model was solved using multi-grid techniques. Results showed that the fluid film thickness of the CoC hip implant was affected by fluid supply conditions: with the increase in the supplied fluid layer, the lubrication film thickness approached to that of the fully blooded solution; when the available fluid layer reduced to some level, the fluid film thickness considerably decreased with the supplying condition. The above finding provides new insights into the lubrication performance of hip implants.

## 1 Introduction

Hip arthroplasty has shown excellent outcomes in decreasing pain and restoring function in patients with degenerative hip joint diseases. The ceramic-on-ceramic (CoC) hip implant is increasingly used due to its outstanding tribological and biocompatible properties. Lubrication plays an important role in the clinical performance of the CoC hip implant. First, poor lubrication is one potential reason of squeaking noise, which is an audible phenomenon receiving increasing concerns (Jarrett et al., 2009), of CoC hip implants (Chevillotte et al., 2010). Second, under deprived lubrication conditions, such as edge loading that occurs when the contact patch between the acetabular and femoral components extends over the rim of the cup, wear of CoC hip bearings significantly increases (Al-Hajjar et al., 2013). In return, third body particles can disrupt lubrication and cause higher friction and squeaking (Sariali et al., 2010). Additionally, poor lubrication increases friction of hip implants which itself can cause loosening (Bishop et al., 2013). Therefore, understanding the lubrication mechanism of CoC hip implants is extremely important.

However, all the previous lubrication analyses of CoC hip bearings were based on an assumption that synovial fluid is sufficiently supplied to the lubricated contact area (Jin et al., 1997; Mabuchi et al., 2004; Meng et al., 2013). This is not always true since realistic conditions may limit the amount of synovial fluid supplied to the contact area of the implant. For example, it has been reported that the volume of the synovial fluid varies much between individuals, ranging from 0.7 to 11.6 mL (Moss et al., 1998). Then it is possible that the available amount of the synovial fluid itself is not sufficient to build up the fluid film. Moreover, under some adverse conditions, such as the edge loading, the inlet distance of fluid may be considerably reduced, which in return will cause starvation. Furthermore, under normal walking or running, hip replacements experience continuously reciprocating motion. Such a repeated reciprocation changes the inlet and outlet of the lubricated contact of hip replacements. Since the film thickness at the outlet of lubricated contact tends to be very small (similar to that in the contact area), when the outlet becomes the inlet, starvation may occur. However, the lubrication performance of CoC hip bearings under starved conditions has not been studied and is thereby still not clear. Therefore, the aim of this study was to investigate the lubrication performance of the CoC hip implant under starved conditions.

## 2 Materials, model and methods

### 2.1 Materials

**Fig. 1:**
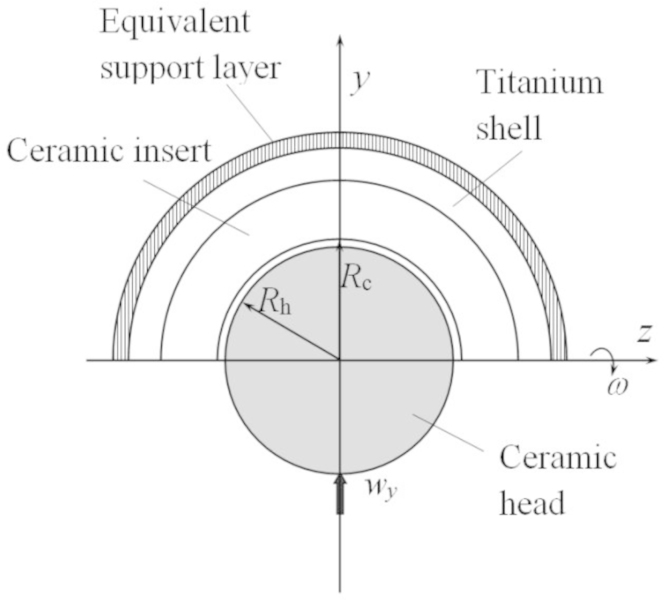
A ball-in-socket configuration for the starved lubrication analysis of the CoC hip implant.

A typical CoC total hip replacement bearing, which consists of three components, a titanium acetabular shell, a ceramic insert and a ceramic head, was considered. The ceramic insert is normally fixed in the titanium acetabular shell using a taper locking mechanism. The initial stability of the acetabular shell is achieved using either cemented or uncemented methods while the long term fixation is reached by the in-growth of bone onto and around the porous-coated shell surface. The spherical ceramic head articulates against the hemi-spherical inner surface of the ceramic insert to form a joint. In the present study, the insert was assumed to be securely fixed to the shell. A uniform thickness of 10 mm and 4 mm was adopted for the ceramic insert and the titanium shell, respectively. The bone and the fixation of the shell were represented by an equivalent support layer with a thickness of 2 mm and appropriate material properties (Jagatia and Jin, 2001). Such a CoC hip bearing configuration is shown in Fig. 1. All the materials of the implant were assumed to be homogeneous and linear elastic. The material properties adopted in the present study are summarized in Table 1. The radius of the head was 14 mm. To achieve a good convergence, the radial clearance was assumed to be 10 µm, which is the lower limit of the radial clearance used for CoC hip bearings (Di Puccio and Mattei, 2015).

The synovial fluid in artificial hip joints behaves as a powerful non-Newtonian fluid under relatively low shear rates. However, under higher shear rates likely to be experienced in the hip joint (10^5^/s), it was reasonable to assume the synovial fluid as Newtonian, isoviscous and incompressible (Cooke et al., 1978; Jin, 2006; Wang et al., 2008; Yao et al., 2003). A realistic viscosity of 0.0025 Pa s was adopted for the synovial fluid in the present study (Yao et al., 2003).

### 2.2 Model

As the first step attempting to investigate the lubrication performance of the CoC hip implant under starved conditions, only the stead-state condition was considered in the present study. The hip joint is generally subjected to three-directional dynamic load and speed during walking. However, the major load and motion components are in the vertical and flexion/extension direction, respectively. Therefore, only the vertical load and the flexion/extension rotation were considered in the present study. The flexion/extension velocity and vertical load were chosen as 2 rad/s and 1500 N, respectively. Both were approximately the average values during a gait (Jin, 2006). Following the previous starved lubrication studies on circular or elliptical contacts (Chevalier et al., 1998; Wijnant, 1998; Yin et al., 2009), it was assumed that a layer of synovial fluid was supplied in the inlet region. The fluid supply condition was represented by the thickness of this inlet fluid layer.

The governing equations of the lubrication model were established in spherical coordinates (Meng, 2013). The starved lubrication was described using a modified Reynolds equation (Meng, 2013; Wijnant, 1998):

(1) $$\sin\;\theta \frac{\partial }{\partial \theta }\left(h^{3}\;\sin\;\theta \frac{\partial p}{\partial \theta }\right)+\frac{\partial }{\partial \varphi }\left(h^{3}\frac{\partial p}{\partial \varphi }\right)=6\eta R_{h}^{2}\omega \sin^{2}\theta \frac{\partial (\theta_{f}h)}{\partial \varphi }$$

where *p* is film pressure; *h* is film thickness; *R*_h_ is the radius of the head; *η* is the viscosity of the periprosthetic synovial fluid; *ω* is the angular velocity of the femoral head; *ϕ* and *θ* are the spherical coordinates (Meng et al., 2010; Meng et al., 2013), and *θ*_f_ is fractional film content. The fractional film content was defined as the ratio between the thickness of the fluid layer (*h*_fluid_) and the gap height (*h*) (Wijnant, 1998):

(2) $$\theta_{f}(\varphi ,\theta )=\frac{h_{\mathrm{fluid}}(\varphi ,\theta )}{h(\varphi ,\theta )}$$

Hence, if the lubricant only partly fills the gap (i.e. the starved region), 0<*θ*_f_<1; whereas if it completely fills the gap (i.e. the pressurized region), *θ*_f_=1.

Besides the boundary conditions similar to the fully flooded lubrication (Meng, 2013):

(3) $$\begin{array}{l} p(0,\theta )=p(\pi ,\theta )=p(\varphi ,0)=p(\varphi ,\pi )=0\\ p\left(\varphi ,\theta \right)\geq 0,0<\varphi <\pi ,0<\theta <\pi \end{array}$$

the following complementary conditions must be fulfilled to obtain a unique solution (Wijnant, 1998):

(4) $$p(\varphi ,\theta )[1-\theta_{f}(\varphi ,\theta )]=0,\;\mathrm{with}\;p(\varphi ,\theta )\geq 0\;\mathrm{and}\;0<\theta_{f}(\varphi ,\theta )\leq 1$$

since a point is either in a pressurized region (*p*>0 and *θ*_f_=1) or in a starved region (*p*=0 and *θ*_f_<1).

The total gap equation consisted of the undeformed gap and the elastic deformation of bearing surfaces due to the film pressure:

(5) $$h=c-e_{x}\;\sin\;\theta \;\cos\;\varphi -e_{y}\;\sin\;\theta \;\sin\;\varphi +\delta$$

where *c* is the radial clearance between the ceramic insert and head (*c*=*R*_c_–*R*_h_; *R*_c_ is the radii of the insert); *e*_*x*_ and *e*_*y*_ are eccentricities of head relative to the cup; *δ* is the elastic deformation of the bearing surfaces, determined by the deformation coefficients of the bearing surfaces and the film pressure.

In addition, the external load components were balanced by the integration of the film pressure:

(6) $$f_{x}=R_{h}^{2}\int_{0}^{\pi }\int_{0}^{\pi }p\sin^{2}\theta \;\cos\;\varphi \;d\theta \;d\varphi =0f_{y}=R_{h}^{2}\int_{0}^{\pi }\int_{0}^{\pi }p\sin^{2}\theta \;\sin\;\varphi \;d\theta \;d\varphi =w_{y}f_{z}=R_{h}^{2}\int_{0}^{\pi }\int_{0}^{\pi }p\;\sin\;\theta \;\cos\;\theta \;d\theta \;d\varphi =0$$

### 2.3 Method

The numerical approach was not very much different from the fully flooded lubrication problem (Meng et al., 2009, 2013). The governing equations were non-dimensionalised to improve the stability of the numerical process. The Reynolds equation was solved using a multi-grid method (Gao et al., 2007; Venner and Lubrecht, 2000). The elastic deformation was calculated using a multi-level multi-integration technique (Gao et al., 2007; Venner and Lubrecht, 2000). The load balance was satisfied through adjusting the eccentricities of the head according to the calculated load components from the hydrodynamic pressure. The deformation coefficients used to calculate the elastic deformation of the bearing surfaces caused by the hydrodynamic pressure were calculated using a finite-element-based method (Wang and Jin, 2004). Three levels of grid were used in the multi-grid solver. On the finest level, 257 nodes were arranged in both the *θ* and *ϕ* directions (Liu et al., 2006).

**Fig. 2:**
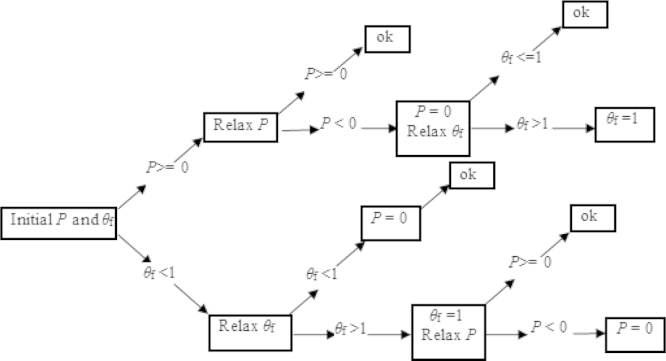
The flowchart of *P* and *θ*_f_ iteration.

However, an additional step had to be performed to calculate the fractional film content, *θ*_f_, and to satisfy the complementary condition. The iteration procedure used by Wijnant (1998) was adopted in this study to stabilize the numerical process. Briefly, the instability caused by the swap between different solutions of *p* and *θ*_f_ was cured by an immediate relaxation on the new variable. For example, after one relaxation of *p* at a given node, if *p*<0, the pressure was set to zero and a new approximation of *θ*_f_ was calculated. If the new updated *θ*_f_>1, its value was set to 1. On the other hand, if a new approximation *θ*_f_ exceeded unity, its value was set to 1 and a new *p* was calculated. The flowchart of *p* and *θ*_f_ iteration is shown in Fig. 2.

## 3 Results

**Fig. 3:**
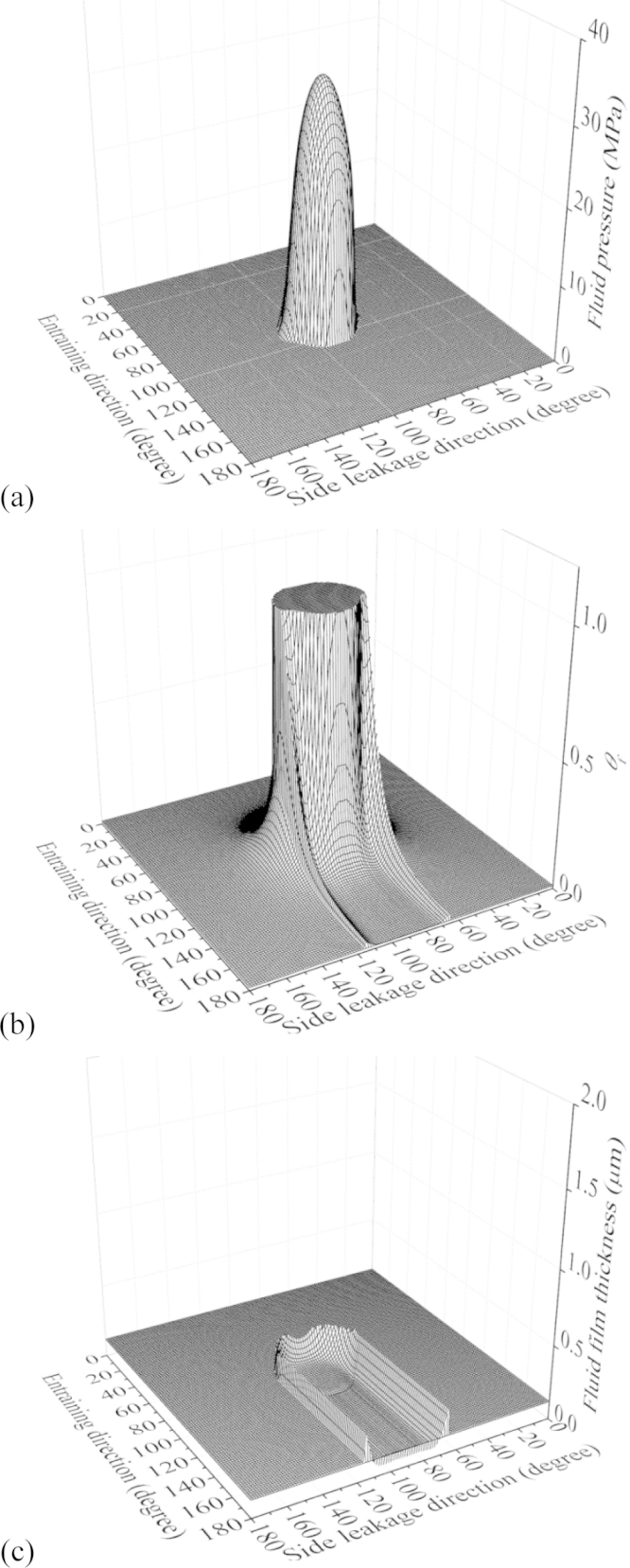
The three-dimensional distributions for the fluid pressure *p* (a), fractional film content *θ*_f_ (b), and fluid film thickness *h*_f_ (c) (*h*_fluid_=0.12 μm).

**Fig. 4:**
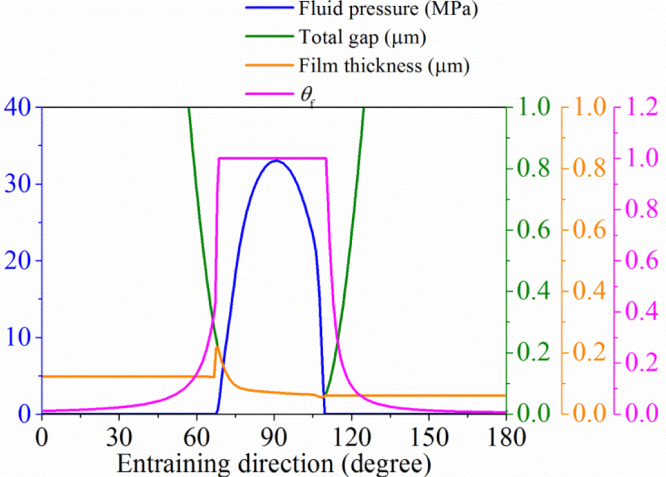
The fluid pressure, total gap, film thickness and fractional film content along the entraining direction (*h*_fluid_=0.12 μm).

The typical three-dimensional distributions of the fluid pressure, fractional film content and fluid film thickness of the CoC hip implant under starved conditions are shown in Fig. 3. The corresponding fluid pressure, total gap, fluid film thickness and fractional film content along the entraining direction are plotted in Fig. 4. The pressurized (*p*>0) and starved (*p*=0) regions can be found in Figs. 3(a) and 4. It is clear in Figs. 3(b) and 4 that in the pressurized region, *θ*_f_=1; while in the starved region, 0<*θ*_f_<1. In addition, the fractional film content had a jump at the inlet meniscus, which was at the same location where the fluid started pressurizing (Figs. 3(a), (b) and 4). Correspondingly, the fluid film thickness also had a jump at the same boundary (Figs. 3(c) and 4). These observations were consistent with the complementary condition. Moreover, the comparison between the fluid film thickness and total gap (Fig. 4) showed that within the pressurized region (*p*>0 and *θ*_f_=1), the fluid film thickness was the same as the total gap, while out of the pressurized region (*p*=0 and 0<*θ*_f_<1), the fluid film thickness was less than the total gap. This was consistent with the definition of *θ*_f_. The above consistencies suggest that the model and solution in the present study are reasonable.

**Fig. 5:**
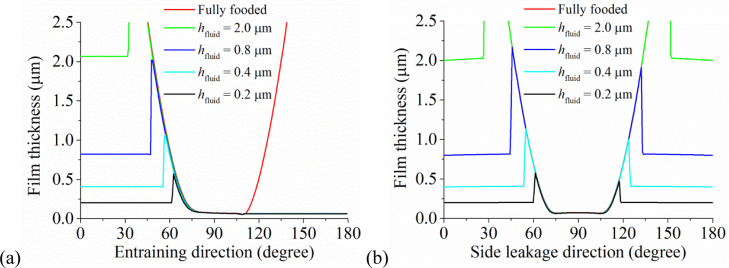
The effect of increasing the thickness of the supplied fluid layer on the fluid film thickness: (a) along the entraining direction and (b) along the leakage direction.

**Fig. 6:**
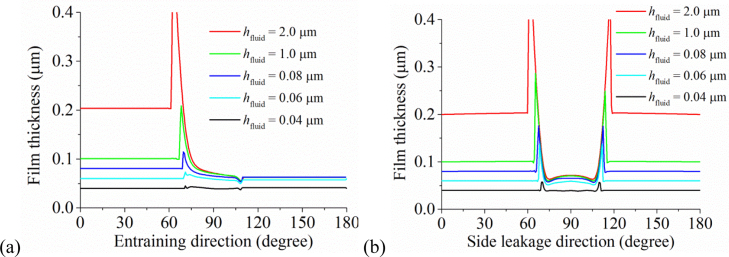
The effect of reducing the thickness of the supplied fluid layer on the fluid film thickness: (a) along the entraining direction and (b) along the leakage direction.

**Fig. 7:**
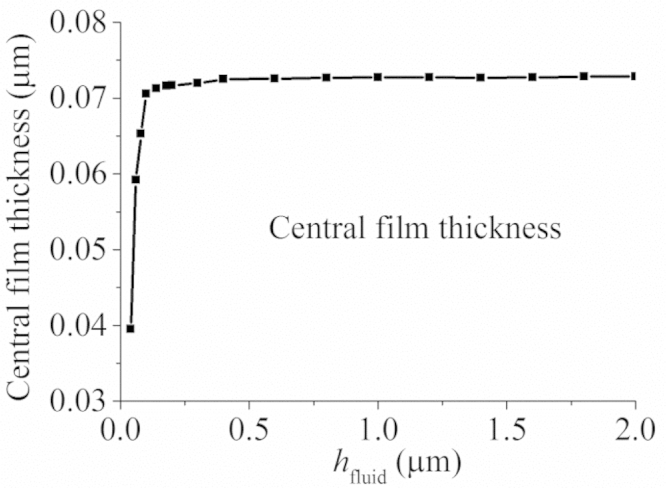
The variation in the central film thickness with the thickness of the inlet fluid layer

The effect of increasing the effective supplied fluid layer on the fluid film thickness is shown in Fig. 5, while the effect of reducing is in Fig. 6. For the cases presented in Fig. 5, the difference in the film thicknesses within the pressurized region was not remarkable except the position of pressurization. Moreover, with the increase in the effective fluid layer, the film thickness approached to that of the fully blooded solution (Fig. 5). Indeed, the central film thicknesses for *h*_fluid_=0.2 µm, *h*_fluid_=2.0 µm and the fully flooded condition were 0.072, 0.073 and 0.075 µm, respectively (Fig. 7). When the effective thickness of the supplied fluid layer reduced to some level (approximately *h*_fluid_=0.14 µm in this study), the fluid film thickness considerably decreased with the fluid supply condition (Fig. 6). The central film thicknesses for *h*_fluid_=0.04 µm reduced to only 0.04 µm (Fig. 7).

## 4 Discussion

The aim of the present study was to investigate the lubrication performance of the CoC hip implant under starved conditions. The results indicated that under severely starved conditions, the fluid film thickness considerably decreased with the supplied fluid layer (Figs. 6 and 7). Moreover, the starved lubrication of the CoC hip implant is very efficient. Under the conditions investigated in this study, even if an effective fluid layer of only 0.2 µm was supplied at the inlet, the film thickness was very close to that of the fully flooded condition (Fig. 5). The implication is that for the steady state conditions considered in this study, only a small amount of fluid is required to achieve a lubricated condition similar to the fully flooded lubrication. However, considering the individual difference between the volumes of capsules, it seems impossible to accurately estimate the required volume of fluid to ensure such a 0.2 µm fluid layer at the inlet of the hip bearings. Moreover, under severely starved conditions the lubrication of the CoC hip implant is also very efficient. For the case of *h*_fluid_=0.04 µm, the central film thicknesses was also 0.04 µm (Fig. 7). The fluid film profile (Fig. 6) showed that under such severely starved conditions, all the supplied fluid was entrained to the contact region and almost no side leakage occurred.

Although the present study appears to be the first study on the starved lubrication of hard–hard hip bearings, the starved lubrication of point or elliptical contacts has been extensively studied under different conditions (Chevalier et al., 1998; Damiens et al., 2004; Venner et al., 2008; Wang and Kaneta, 2007; Wijnant and Venner, 1999; Yin et al., 2009). Therefore, a comparison between the present study and these studies is helpful for assessing the validity of the present study. Indeed, the above findings are consistent with those of the starved point contact lubrication (Chevalier et al., 1998). Such an agreement provides confidence that the results in the present study are reasonable.

It should be pointed out that the decrease in the film thickness under severely starved conditions has important clinical implications for CoC hip implants since a reduction in film thickness will cause squeaking noise and increase wear. Therefore, understanding how to improve the tribological performance of CoC hip implants under severely starved lubrication may lead to improved hip implants. Moreover, the practical conditions that may cause starvation, such as the inlet distance and repeated reciprocating motions, are mainly related to loading and kinetic conditions and geometric designs. In order to explore how to improve the tribological performance of CoC hip implants under severely starved lubrication, more realistic three-dimensional time-dependent loading and kinetic conditions, adverse conditions (such as edge loading), and geometric parameters should be analyzed in the future.

Lubrication analysis has played an important role in the development of hip implants through investigating how the geometric parameters and working conditions affect the fluid film thickness (Dowson et al., 2004; Jin et al., 1997; Mattei et al., 2011). However, all the previous lubrication analyses of hip bearings were based on the assumption that synovial fluid is sufficiently supplied. Since starved conditions theoretically exist in hip implants, the lubrication performance of other hip bearings under starved conditions is equally important. In such context, the model and results presented in this study are important not only for the CoC hip implant, but also for other hard–hard hip bearings.

There are, of course, limitations in the present study as discussed above. For example, as the first step towards investigating the starved lubrication of CoC hip implants, only the steady state condition and one group of loading and geometric parameters were considered. Moreover, to achieve a good convergence, a smaller radial clearance was used. Despite the above limitations, the present study has paved the way towards fully understanding lubrication performance of the CoC hip implant under starved lubrication and provided important initial understanding.

## 5 Conclusions

A starved lubrication model was presented and solved for the CoC hip implant for the first time. Results showed that for the conditions considered in this study, the lubrication performance of the CoC hip implant was affected by fluid supply: with the increase in the supplied fluid layer, the lubrication film thickness approached to that of the fully blooded solution; when the available fluid layer reduced to some level, the fluid film thickness considerably decreased with the reduction of fluid supplied to the inlet. Such a variation in the fluid film thickness with starved conditions implies the importance of considering starved lubrication in the CoC hip implant.

## Figures and Tables

**Table 1:** Material properties of titanium shell, ceramic and equivalent support layer used in the present study.

	Elastic modulus (GPa)	Poisson׳s ratio
Titanium	110	0.3
Equivalent support layer	2.27	0.23
Ceramic	380	0.26
